# Parenting Styles as a Moderator of the Association between Pubertal Timing and Chinese Adolescents’ Drinking Behavior

**DOI:** 10.3390/ijerph19063340

**Published:** 2022-03-11

**Authors:** Hui Ling, Yaqin Yan, Hong Feng, Amin Zhu, Jianren Zhang, Siyang Yuan

**Affiliations:** 1 Psychology Department, Hunan Normal University, Changsha 410081, China; yanyaqin_hnnu@126.com (Y.Y.); aminzhu_phychology@163.com (A.Z.); jianrenzhang_hnnu@126.com (J.Z.); 2 Department of Student Affairs, Hunan First Normal University, Changsha 410205, China; 3 Research Institute of Education, Hunan Wenjin Education Group, Changsha 410031, China; fenghong@wjjyedu.onexmail.com; 4 School of Dentistry, University of Dundee, Dundee DD1 4HN, UK; s.z.yuan@dundee.ac.uk

**Keywords:** puberty timing, parenting styles, drinking behavior

## Abstract

Background: Previous work has indicated that pubertal timing and parenting styles are associated with adolescents’ drinking behavior, but studies on the relationship between the above three variables are lacking. Methods: Participants were 1408 Chinese adolescents aged 11–16 years old (46.52% girls). The data emphasized pubertal timing, parenting styles, drinking behavior, and socioeconomic and demographic characteristics of the adolescent and his or her family. Results: Early pubertal timing was related to drinking behavior; however, parenting styles played a moderating role. For male adolescents, father emotional warmth, mother rejection, and mother emotional warmth moderated the relationship between early pubertal timing and drinking behavior. For female adolescents, mother rejection, mother emotional warmth, and mother over-protection moderated the relationship between pubertal timing and drinking behavior. Conclusions: Parenting styles that include emotional warmth, rejection, and over-protection appear to influence the negative outcomes associated with early pubertal timing, and may be useful in reducing adolescents’ drinking behavior.

## 1. Introduction

Puberty is the transition from childhood to adulthood. As a critical developmental milestone, puberty is notable for its rapid and near-simultaneous transformation across biological, social, and psychological domains. As physical appearance matures, individuals often necessitate a reorganization of identity and self-perception [1]. Pubertal timing refers to individual differences of puberty development time [2]. The pubertal timing of individuals can be categorized as early, moderate, or late, depending on the time of pubertal developments compared with those of a reference group [3]. Early puberty makes the adolescent seem more physically mature than their peers, but this is not paralleled with psychosocial maturity. Even if these adolescents have reached sexual maturity, it does not mean that they reached adulthood in psychosocial terms.

Pubertal timing is influenced by both genetic and environmental factors [4]. The major pubertal changes usually appear from age 8 to 14, occurring at age 10 (typically after age 8 for girls and age 9 for boys); although, this range is very broad, and may vary culturally [5]. In China, the average age at which puberty begins has decreased compared with the national epidemiological survey data in 2005. The puberty timing for boys (10.1 years old) and girls (8.5 years old) was 0.4 and 0.7 years earlier, and the menarche age for girls (12.1 years old) was 0.1 years earlier in 2020 [6]. Scholars acknowledge that early pubertal timing is related to many internalizing problems (depression, anxiety, somatization symptoms, etc.), and externalizing behavior problems (aggression, drinking behavior, substance use, decline of academic achievement, etc.) in adolescence and even early adulthood [2,7,8,9,10].

Contextual amplification theory posits that the interaction of contextual factors (including family environment) and pubertal timing will affect the risk of problem behaviors among adolescents. This theory holds that the family environment plays a moderating role between pubertal timing and externalized problem behavior, and emphasizes the interaction between the family environment and pubertal timing. Family environment, as an important contextual factor for adolescent development, can be regarded as both a risk factor and a protection factor [11]. If the family environment of teenagers is “strict and inconsistent”, they will be more prone to externalization problems [12]. On the contrary, if the family environment of teenagers is more supportive and beneficial, the risk of problem behavior will be reduced, in which the supportive contextual factors play a certain protective role for teenagers. Existing studies show parenting practices as a moderator between early pubertal timing and substance use [13]. Yet, little is known regarding the role of parenting styles in the relationship between pubertal timing and alcohol drinking. Alcohol use is a direct threat to adolescent health, and it creates risks of around 230 different identified health outcomes in the form of disease, injury, or mental health problems [14,15,16]. Recent studies found that adolescence alcohol use is linked to suicidal ideation and suicide attempts [17,18], gambling, abnormal fear-related behavior, and alcohol use disorders [19,20]. Alcohol use and abuse among young people (under 21 years of age) is a pervasive problem worldwide [21]. In China, alcohol drinking is more common than other substance use behavior among adolescents [18], given the fact that alcohol drinking is more culturally acceptable in China. Existing studies indicate that teenagers’ drinking behavior is becoming increasingly serious, and teenagers’ heavy drinking and drunken behavior has also increased. In particular, the age at which teenagers start drinking shows a downward trend [22].

Existing research indicates that early pubertal timing and parenting styles might influence drinking behavior in youth. Adolescence is a time of vulnerability and adjustment, when impulse control is still relatively immature. The risk of adolescence drinking is positively associated with authoritative, disengaged, or harsh parenting; parental provision of alcohol; and favorable attitudes towards alcohol use and drinking/misuse [23]. On the other hand, it is negatively associated with parental monitoring, support, and democratic parenting in the framework of a high-quality parent–child relationship [24]. Usually, harsh parenting in childhood, including negative ways of rejection, punishment, and abuse, have a direct impact on early pubertal timing and girls’ substance abuse behavior (such as drinking) [25]. However, no interaction between the three factors has been reported [26]. In addition, positive parenting in childhood may reduce the possibility of early pubertal timing in boys and girls [27]. Other studies have shown that the risk of early maturing boys and girls trying to drink is much higher than that of non-early maturing boys and girls, and parental monitoring can reduce the drinking behavior of boys and girls who mature early [2]. However, studies on the relationship between pubertal timing, parenting styles, and drinking behavior are lacking. Therefore, the primary objective of the present study is to investigate the relationships among parenting styles, pubertal timing, and adolescents’ drinking behaviors.

Evidence suggests that cultural norms might influence the relationship between parenting styles and behavior problems in youth [28,29]. Examination showed that among English-speaking Mexican Americans, mother acceptance and hostile control were not correlated; among Spanish-speaking Mexican Americans, mother acceptance and hostile control were positively related; and among Euro-Americans, mother acceptance and hostile control were negatively related. By collecting data from a predominantly collectivist culture such as Chinese [30], this study hopes to contribute to understanding the impact of culture on parenting and teenagers’ behavioral problems. This is the second objective of the present study.

There were gender differences of the relationship between pubertal timing and adolescent drinking behavior. Girls who mature early are twice as likely to try drinking as those who mature with average timing or relatively late. Girls who mature early had a higher rate of drunk behavior than girls who mature with average timing or relatively late. Association with deviant peers is the intermediary variable between girls who mature early and drinking, whereas parental monitoring is the protective factor [2]. However, for boys, the relevant results are not consistent. Early studies believe that early pubertal timing benefits boys because peers usually view them as calm, having good character, and being more sophisticated, whereas late maturity is a risk factor for social and psychological adaptation, as well as mental health, which will increase boys’ adaptation problems [31]. However, recent studies assert that early pubertal timing in boys correlates with a higher risk of substance use, illegal acts, and a higher drunk rate [32]. Therefore, the third objective of the study is to investigate the different mechanisms through which parenting styles influence the relationship between pubertal timing and drinking behavior in male and female adolescents. 

## 2. Materials and Methods

### 2.1. Participants

We recruited a total of 1408 adolescents, all of whom were between 11 and 16 years old. A cluster sampling method was performed to choose classes from eight middle schools in Changsha, Yueyang, Hengyang, and Huaihua in the Hunan province of China, totaling 1540 students as potential participants. There were no immigrants in the sample.

### 2.2. Measures

#### 2.2.1. Demographic Information

Demographic information for the study was obtained by questionnaire questions, including age, gender, grade, school, residential address, parental marital status, parents’ education level and occupation, and number of siblings.

#### 2.2.2. Pubertal Timing

Pubertal timing was measured by the Pubertal Development Scale (PDS). This scale is a noninvasive self-report measure of pubertal development. It consists of five items: age at menarche, breast growth in girls, deepening-voice and facial hair growth in boys, body hair growth, and growth spurt and skin changes (especially pimples) in both genders. Response options were: “not yet started” (1 point), “barely started” (2 points), “definitely started” (3 points), or “seems finished” (4 points). In addition, females were asked about menarche; either no (0 point) or yes (1 point). A higher PDS total score indicates earlier pubertal timing among peers. This scale has good validity and reliability, with Cronbach’s α ranging from 0.68 to 0.83, and in this study, it was 0.681. Chan and colleagues [33] translated and revised the Pubertal Development Scale based on the Chinese adolescent population. Their translated scale indicates a Kendall τ–b index between self-reported PDS and evaluator PDS as 0.61 in females and 0.49 in males. 

#### 2.2.3. Parenting Style

Parenting style was measured using the Short-form Egna Minnen av Barndoms Uppfostran for Chinese (s-EMBU-C). This scale was translated and revised from Egna Minnen av Barndoms Uppfostran (EMBU) [34] by Jiang and colleagues, based on Chinese college students [25]. The scale is reported by adolescents, and includes 21 father items and 21 mother items. Each item was measured on a 4-point Likert scale: “never” (1 point), “occasional” (2 points), “often” (3 points), and “always” (4 points). Three dimensions of parenting style have been determined as rejection, emotional warmth, and over-protection. This questionnaire has relatively good validity and reliability, with Cronbach’s α ranging from 0.74 to 0.84, and in this study, it was 0.864. Test–retest reliability ranged from 0.70 to 0.81 [35].

#### 2.2.4. Drinking Behavior

Drinking behavior was measured using the Adolescents’ Drinking Behavior Surveillance System, which refers to the Youth Health Risk Behavior Surveillance System (2005). It has 3 items: the first item was “How many days have you drunk more than 1 glass in the past 30 days?”; the second item was “How many days have you drunk more than 5 glasses in the past 30 days?”; The third item was” How many days have you been drunk in the past 30 days?”. The answers were: (1) never; (2) 1–2 days; (3) 3–5 days; (4) 6–9 days; (5) 10–19 days; and (6) more than 20 days. Responses were categorized into “no” (response 1) or “yes” (2, 3, 4, 5, and 6). This categorization method was shown to be effective in revealing adolescents’ drinking situations in the existing study [36].

### 2.3. Procedure

Prior to study participation, all participants gave written informed consent, including written parental consent. The study was approved by the Ethical Committee for Scientific Research in the Hunan Normal University. Surveys were conducted in June 2021. Student participation in the survey was voluntary. Surveys were administered during class time by the research staff. To ensure confidentiality, all questionnaires were completed anonymously. Participants were told that if they were uncomfortable with the answers, they could choose not to respond to the questionnaire or any of the questions on the questionnaire.

### 2.4. Data Analysis

All analyses were conducted in SPSS version 22.0 (SPSS, Inc., Chicago, IL, USA). Respondents who answered “yes” in the first item (drunk more than 1 glass in the past 30 days) are considered as “tried drinking individuals”; those who answered “yes” in the second item (drunk more than 5 glasses in the past 30 days) are considered as “heavy drinking individuals”; whereas those who answered “yes” in the third item (have been drunk in the past 30 days) are considered as “drunken individuals”. Twelve groups were identified based on gender and age. Three perceived pubertal timing groups were defined, consistent with prior descriptions in the literature: Early (>1 SD above the mean for the same age and sex peers), Average (within 1 SD from the mean), and Late (>1 SD below the mean) [37]. Table 1 shows the percentage of participants in each puberty stage by gender and age level.

Firstly, descriptive statistics were used to report the frequencies. Secondly, chi-squared tests and Pearson correlations were used to investigate individual differences in main variables and the association between pubertal timing, drinking behavior, and parenting styles. Finally, hierarchical linear regression analyses were examined to investigate the direct predictive effect of pubertal timing and parenting styles on adolescent drinking behavior, and parenting style as a moderator of the association between pubertal timing and adolescent drinking behavior. Before testing possible moderation effects, pubertal timing scores were centralized (the mean subtracted from the value) [38].

All data were reported by the adolescents in the current study, and to estimate the common method variance, we used Harman’s single-factor test [39]. Factor analyses were conducted on all items, and found that 18 factors with eigenvalues greater than one were extracted, with Factor 1 accounting for 14.44% of the variance (less than 40%), which suggested that there is no substantial common method variance in this study.

## 3. Results

Complete data were available from 1408 students (753 boys and 655 girls), resulting in a 91.43% response rate. In terms of demographic data, 35.72% of the participants came from the city (*n* = 503), whereas 64.28% of them came from rural areas (*n* = 905).

Table 2 presents the frequency (%) of the characteristics of adolescents by their drinking status and results of association (*n* = 1408), as well as pubertal timing differences in drinking groups. During the month preceding the study, among boys, the tried drinking rate was 32.01%, the heavy drinking rate was 13.55%, and the drunken rate was 7.84%. Among girls, the tried drinking rate was 23.51%, the heavy drinking rate was 7.02%, and the drunken rate was 3.36%. Moreover, there were gender differences (*χ*^2^ = 11.20, *p* < 0.01; *χ*^2^ = 15.85, *p* < 0.001; *χ*^2^ = 12.95, *p* < 0.001), grade differences (*χ*^2^ = 16.87, *p* < 0.001; *χ*^2^ = 19.35, *p* < 0.001; *χ*^2^ = 7.57, *p* < 0.05), and hometown differences (*χ*^2^ = 16.80, *p* < 0.001; *χ*^2^ = 10.50, *p* < 0.01; *χ*^2^ = 6.82, *p* < 0.01) within the different levels of drinking. Table 3 and Table 4 present the pubertal timing differences on drinking groups. (*χ*^2^ boys = 6.94, *p* < 0.05; *χ*^2^ boys = 23.58, *p* < 0.001; *χ*^2^ boys = 12.80, *p* < 0.01; *χ*^2^ girls = 22.19, *p* < 0.001; *χ*^2^ girls = 31.01, *p* < 0.001; *χ*^2^ girls = 14.49, *p* < 0.01).

A higher PDS total score indicates earlier pubertal timing among peers. There are four levels of the adolescents’ drinking behavior in the past 30 days: “0” indicates there is no drinking behavior at all; “1” indicates one type of drinking behavior (tried drinking behaviors, heavy drinking behaviors, or drunken behaviors); “2” means two drinking behaviors; and “3” means three drinking behaviors. Therefore, a higher score indicates more drinking behaviors [36].

Bivariate correlations among primary study variables are reported in Table 3. As shown, boys’ PDS was significantly and positively related to tried drinking behaviors, heavy drinking behaviors, and drunken behaviors, whereas it was not significantly related to parenting styles. Father rejection and mother rejection were significantly and positively related to boys’ drinking behaviors. Father emotional warmth and mother emotional warmth were significantly and negatively related to boys’ drinking behaviors. Moreover, girls’ PDS was significantly and positively related to girls’ drinking behaviors, mother rejection, and mother over-protection. Father rejection, father over-protection, mother rejection, and mother over-protection were significantly and positively related to girls’ drinking behaviors. Mother emotional warmth was significantly and negatively related to girls’ drinking behavior; however, father emotional warmth was only significantly and negatively related to girls’ tried drinking behaviors.

Hierarchical linear regression analyses were conducted to evaluate the moderating effects of parenting styles (measured by centralized dimension scores) between pubertal timing (measured by centralized PDS total scores) and adolescents’ drinking behavior.

**Figure 1:**
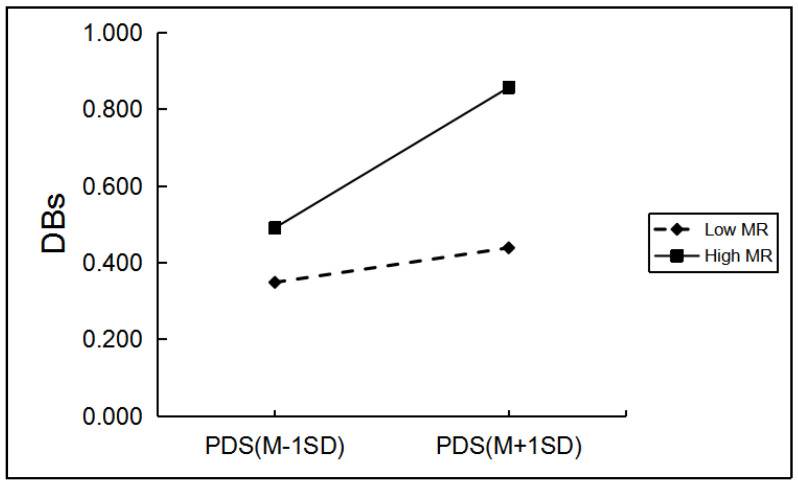
The two-way interaction between PT and MR in predicting boys’ DBs. Note: PDS, the total score of pubertal development scale; MR, mother rejection; DBs, drinking behaviors.

**Figure 2:**
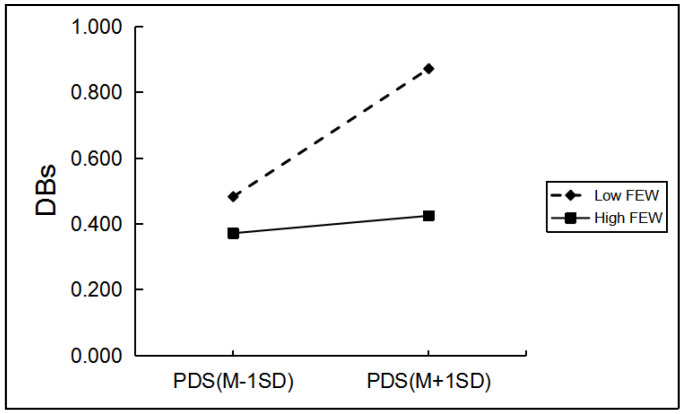
The two-way interaction between PT and FEW in predicting boys’ DBs. Note: PDS, the total score of pubertal development scale; FEW, father emotional warmth; DBs, drinking behaviors.

**Figure 3:**
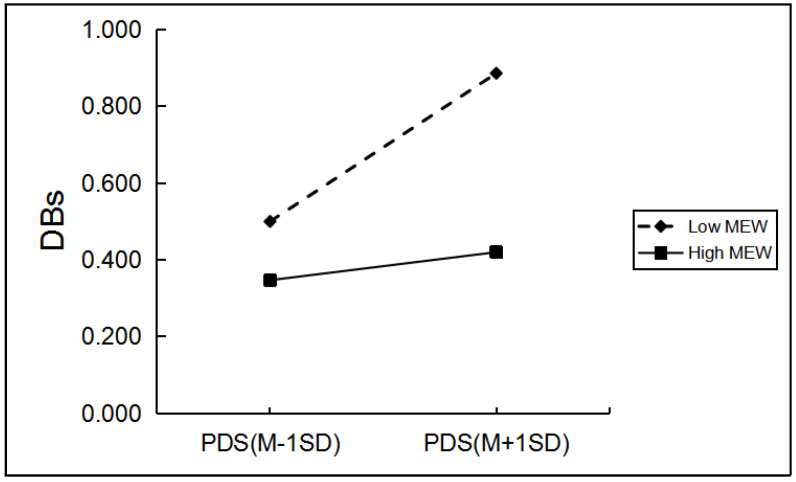
The two-way interaction between PT and MEW in predicting boys’ SBs. Note: PDS, the total score of pubertal development scale; MEW, mother emotional warmth; DBs, drinking behaviors.

Among boys, grade and hometown can significantly predict boys’ drinking behaviors. When controlling for grade and hometown, as shown in Table 4, only father emotional warmth, mother rejection, and mother emotional warmth significantly moderated the relationship between PDS and boys’ drinking behaviors. Regarding the moderating role of mother rejection, simple slope analyses indicated the effect of pubertal timing on boys’ drinking behaviors was greater for boys with a high level of mother rejection (*β* = 0.18, *p* < 0.001) than for those with a low level of mother rejection (*β* = 0.05, *p* > 0.05; Figure 1). The effect of pubertal timing on boys’ drinking behaviors was greater for boys with a low level of father emotional warmth and mother emotional warmth (*β* = 0.20, *p* < 0.001; *β* = 0.19, *p* < 0.001) than for boys with a high level of father emotional warmth and mother emotional warmth (*β* = 0.03, *p* > 0.05; *β* = 0.04, *p* > 0.05; Figure 2 and Figure 3).

**Figure 4:**
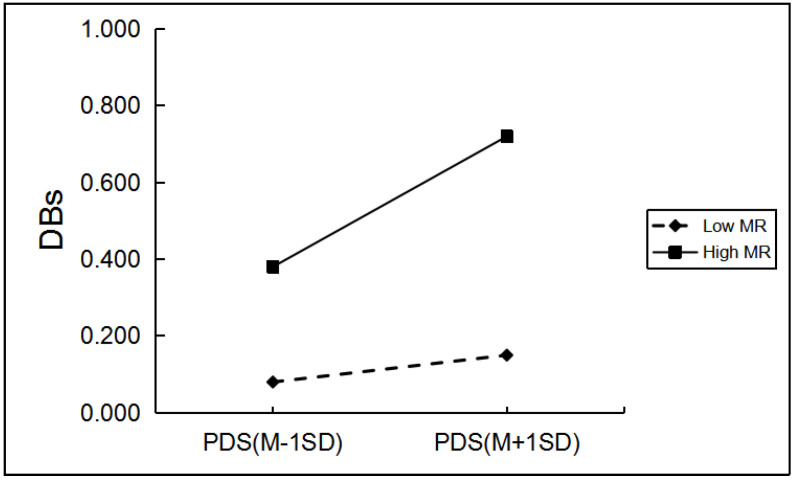
The two-way interaction between PT and MR in predicting girls’ DBs. Note: PDS, the total score of pubertal development scale; MR, mother rejection; DBs, drinking behaviors.

**Figure 5:**
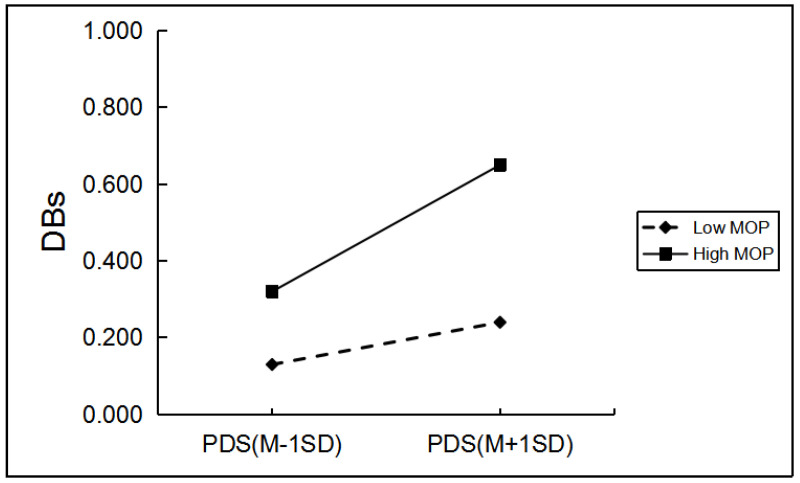
The two-way interaction between PT and MOP in predicting girls’ DBs. Note: PDS, the total score of pubertal development scale; MOP, mother over-protection; DBs, drinking behaviors.

**Figure 6:**
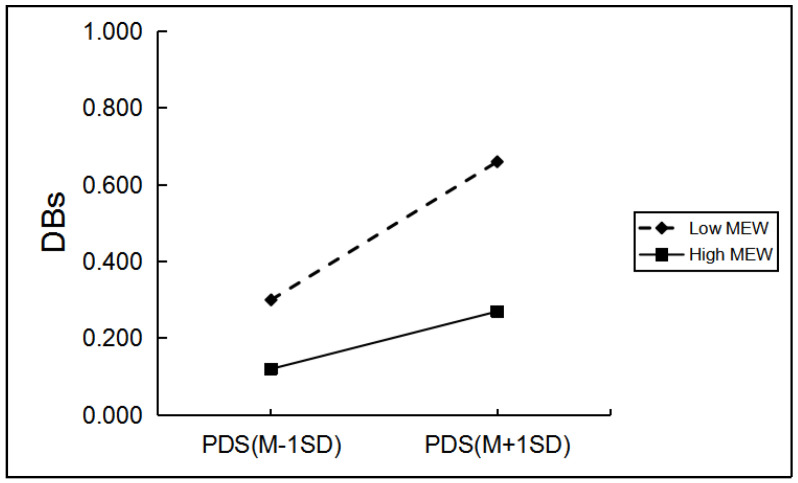
The two-way interaction between PT and MEW in predicting girls’ DBs. Note: PDS, the total score of pubertal development scale; MEW, mother emotional warmth; DBs, drinking behaviors.

Among girls, as shown in Table 4, only mother over-protection, mother rejection, and mother emotional warmth significantly moderated the relationship between PDS and girls’ drinking behaviors. Regarding the moderating role of mother rejection and mother over-protection, simple slope analyses indicated the effect of pubertal timing on girls’ drinking behaviors was greater for those with a high level of mother rejection and mother over-protection (*β* = 0.17, *p* < 0.001; *β =* 0.17, *p* < 0.001) than for girls with a low level of mother rejection and mother over-protection (*β* = 0.04, *p* > 0.05; *β* = 0.06, *p* > 0.05; Figure 4 and Figure 5). In the high (*β* = 0.08, *p* < 0.05) and low mother emotional warmth groups (*β* = 0.18, *p* < 0.001), PDS can significantly and positively predict girls’ drinking behavior (Figure 6). However, compared with the low mother emotional warmth group, PDS in the high mother emotional warmth group had a lower impact on girls’ drinking behavior (Δ*β* = 0.10).

## 4. Discussion

The current study investigates parenting practices as a moderator between early pubertal timing and adolescent drinking behavior. The results show that adolescents’ early pubertal timing correlates with more drinking behaviors, which is consistent with the previous studies [2,32,40]. The gender-intensification hypothesis holds that adolescent peers and adults exert increased socialization pressures on adolescents to conform to traditional notions of masculinity and femininity [41]. Early pubertal timing may accelerate the intensification of sex role attitudes. Drinking is regarded as a masculine behavior [42]. Therefore, boys may believe that drinking makes them more like men. In addition, adolescents yearn for independence and respect; thus, they do things against adult rules and authority, such as delinquent behavior, smoking, and drinking. Early maturing teenagers tend to associate with older teenagers, and have more opportunities to have contact with some adult behaviors, such as drinking. Early maturing teenagers will imitate the behavior of older teenagers.

By exploring the moderation relationship, we identified several explanations for the pubertal-timing–adolescent-drinking association, so we can speak more directly concerning the different perspectives on changing family contexts. We found that for different genders, the mechanism of parenting styles regulating the relationship between puberty timing and adolescent drinking behavior is different.

For boys, paternal emotional warmth and mother rejection moderated the effects of pubertal timing on alcohol use. High paternal emotional warmth could lower the drinking risk in early developing boys, whereas high mother rejection may increase drinking risk in them. This study verified that paternal emotional warmth is an important psychological resource for boys to deal with incidents and social pressure, and the emotional connection with parents and important others can buffer the negative impact of disadvantages to a certain extent. Father emotional warmth felt by boys has a very special significance. On the one hand, it is because fathers play an irreplaceable role in the growth of boys, and affect gender roles, moral development, deviant behavior, social adaptation, and so on [43]. On the other hand, due to the influence of the Chinese traditional parenting style (“strict father and loving mother”), most fathers are strict (punish the child when he/she make mistakes), and pay less attention to showing loving care for their child, whereas the mother provides love and warmth. Therefore, for most children, father emotional warmth is insufficient. Compared with the father, mother rejection will have a greater negative impact on boys.

In female adolescents, mother rejection, mother emotional warmth, and mother over-protection moderated the relationship between pubertal timing and drinking behaviors. Results from simple slope testing show that a low level of mother emotional warmth increases female adolescents’ drinking behavior, regardless of their pubertal timing status. In Chinese traditional culture, compared with fathers, mothers are more responsible for providing love and warmth to their children, and girls feel their mothers’ emotional warmth more than boys [44]. Hence, lower mother emotional warmth would have a greater negative impact on girls. The current study also found that a high level of mother rejection and mother over-protection increase drinking behavior in female adolescents with early pubertal timing. One possible reason is that mother rejection and over-protection would make them experience a psychological defeat, and feel lower mother emotional warmth. In addition, mother protection would make girls feel more pressure and discomfort. Accordingly, it may reduce their self-worth and self-esteem, leading to more drinking behaviors.

Through analyzing the differences between gender and location, it is found that girls’ drinking behaviors are significantly less than that of boys, and the drinking behavior of rural students is significantly higher than that of urban students, which is consistent with the existing results in China [45]. This result is easy to understand in China. Influenced by the concept of gender roles, drinking is usually regarded as a male behavior. Never-drinking boys may be ridiculed by peers, whereas the public holds a negative and disapproving attitude towards women drinking, especially girls. Most people will regard drinking girls as “alternative” or “bad girls”, so they will face more pressure than boys. In addition, some studies have found that girls’ awareness of the harm of drinking is significantly higher than boys, and adolescents’ awareness of the harm of drinking is significantly negatively correlated with their drinking behavior. This study also found that at older ages, the drinking behaviors of teenagers also increased significantly. Drinking behaviors may raise teenagers’ feelings of maturity. Many teenagers claim “I have grown up” or “adults should drink” as their reason for drinking [46]. Teenagers in grade nine have the highest proportion of tried drinking, heavy drinking, and drunken behavior. At this grade, they have to face the pressure of preparing for the high-school entrance exam, which causes teenagers to have stress, anxiety, and other negative emotional experiences. Teens’ expectancies about drinking include beliefs that using alcohol will provide stress relief, facilitate social interaction, or make them feel good [47]. The results suggest that parents and teachers should pay enough attention to teenagers by providing adequate support to adolescents to effectively cope with stress. This may help to discourage adolescents to seek risky behaviors, such as alcohol drinking, to reduce their stress and anxiety.

Rural students have significantly more drinking behaviors than urban students. One reason might be that there are more left-behind children among rural students [48], and they are more prone to drinking behavior due to their lack of education and supervision from parents for long periods of time [49]. Moreover, the caregivers of rural students always have low educational levels, and they do not fully understand the harm of drinking. Many caregivers have the habit of drinking, even in front of children. Many rural teenagers learn to drink from their caregivers [50].

Several of our results are consistent with previous studies. First, the current study shows that compared to on-time pubertal timing adolescents and late pubertal timing adolescents, adolescents with early pubertal timing reported a higher drinking rate [2,30]. Second, parenting styles may affect adolescents’ drinking behaviors [51]. This suggests that when raising their children, parents should adopt a warm, supportive, and inclusive parenting style, and less or no harsh parental practices. At the same time, they should also monitor and restrict their children to establish a good parent–child relationship, which will help to reduce adolescents’ dangerous behaviors.

Our findings should be interpreted in light of a few limitations. First, this study adopts PDS to measure pubertal timing. Although self-assessment of PDS is a well-known, reliable, and valid measure of pubertal status [52], it could be improved by more objective clinician assessments in future, e.g., clinician reports of the Tanner stage. It is recommended that the examination of pubertal staging is best done by palpation and visualization [53]. Second, all measures were based on self-reported questionnaires completed by adolescents in a public setting. Since parenting styles rated by children and their parents are different [54], our self-report measure includes any bias related to adolescents’ perceptions of parenting styles. Future studies may benefit from an explicit focus on variation in adolescent versus parent reports of parenting styles during this developmental period. Third, this sample came from 11–16 year-old Chinese adolescents. Thus, the conclusion may have limited generalizability to other age ranges or other cultural backgrounds. An additional limitation is that this cross-sectional study relied on an assessment from age 11–16, and did not refer to the pubertal timing as a life course experience, but to the current status of sexual maturity. Forth, parents suffering from alcohol-use disorders or dual diagnoses is a significant risk factor for harsh and deficient parenting behavior [55,56,57,58], but this study did not examine this variable. Finally, adolescence is very complex time of life, and other factors besides parenting styles and pubertal timing are involved in its development; therefore, the possibility of omitted variables cannot be excluded (e.g., parental alcohol permissiveness, peer influences, socioeconomic status, immigrants or not, autonomy).

In summary, the findings from this study show that parenting style is a moderator of the association between pubertal timing and Chinese adolescents’ drinking behavior. Future research should continue to unpack the specific influence factors. Subsequent research should also move toward more specificity in the effect of alcohol use on future mental health. Ideally, future research may benefit from repeated adolescent reports over time as a longitudinal study, in order to explicitly examine within-individual change. Further establishing these lines of research will inform the development and dissemination of parenting interventions that directly address the needs of at-risk adolescents, and aid in reducing the impact of early pubertal timing on adolescents’ developmental outcomes.

## 5. Conclusions

Based on prior research and the present results, it may be worth considering variations in pubertal timing and parenting styles for alcohol prevention. Schools, clinical institutions, and parents should realize that pubertal timing is not just an indicator of advanced cognitive, neurological, or social development or maturity. Early pubertal timing teenagers may be exposed prematurely to risks, and thus, need support from parents and other caregivers. Emotional connection with parents or other significant people is essential, which can help reduce the risk of problem behaviors [59,60]. This suggests that parents should adopt a warm, supportive, and inclusive parenting style; appropriately monitor and restrict children; and establish a good parent–child relationship, rather than harsh parenting. The findings of the study also suggest the importance of parenting styles in the context of gender differences, which would be especially beneficial in the understanding of parental emotional warmth in the upbringing of boys and girls.

## Figures and Tables

**Table 1:** PDS categories in groups of genders and ages.

Gender	Age	Early *n* (%)	Average *n* (%)	Late *n* (%)
Boys	11 (*n* = 8)	0 (0.0)	6 (75.0)	2 (25.0)
	12 (*n* = 134)	24 (17.9)	91 (67.9)	19 (14.2)
	13 (*n* = 183)	28 (15.3)	131 (71.6)	24 (13.1)
	14 (*n* = 169)	39 (23.0)	100 (59.2)	30 (18.1)
	15 (*n* = 221)	44 (19.9)	137 (62.0)	40 (18.1)
	16 (*n* = 38)	3 (7.9)	27 (71.1)	8 (21.1)
Girls	11 (*n* = 16)	4 (25.0)	9 (56.3)	3 (18.8)
	12 (*n* = 161)	34 (21.1)	101 (62.7)	26 (16.1)
	13 (*n* = 143)	32 (22.4)	92 (64.3)	19 (13.2)
	14 (*n* = 186)	34 (18.3)	134 (72.0)	18 (9.7)
	15 (*n* = 132)	23 (17.4)	85 (64.4)	24 (18.2)
	16 (*n* = 17)	4 (23.5)	10 (58.8)	3 (17.6)

**Table 2:** Frequency (%) of the characteristics of adolescents by their drinking status and results of association (*n* = 1408), and pubertal timing differences in drinking groups.

		Tried Drinking *n* (%)	Heavy Drinking *n* (%)	Drunken Behavior *n* (%)
Gender	Boys (*n* = 753)	241 (32.01)	102 (13.55)	59 (7.84)
	Girls (*n* = 655)	154 (23.51)	46 (7.02)	22 (3.36)
*χ* ^2^		12.52 ***	15.85 ***	12.95 ***
Grade	Seven (*n* = 341)	75 (21.99)	16 (4.69)	10 (2.93)
	Eight (*n* = 492)	126 (25.61)	52 (10.57)	29 (5.89)
	Nine (*n* = 575)	194 (33.74)	80 (13.91)	42 (7.30)
*χ* ^2^		16.87 ***	19.35 ***	7.57 *
Hometown	City (*n* = 503)	108 (21.47)	35 (6.96)	18 (3.58)
	Rural (*n* = 905)	287 (31.71)	113 (12.49)	63 (6.96)
*χ* ^2^		16.80 ***	10.50 **	6.82 **
Boys	Early (*n* = 138)	57 (41.30)	36 (26.09)	21 (15.22)
	Average (*n* = 492)	145 (29.47)	56 (11.38)	31 (6.30)
	Late (*n* = 123)	39 (31.71)	10 (8.13)	7 (5.69)
*χ* ^2^		6.94 *	23.58 ***	12.80 **
Girls	Early (*n* = 131)	51 (38.93)	25 (19.08)	11 (8.40)
	Average (*n* = 431)	82 (19.03)	12 (2.78)	7 (1.62)
	Late (*n* = 93)	21 (22.58)	9 (9.68)	4 (4.30)
*χ* ^2^		22.19 ***	31.01 ***	14.49 **

Note: * *p* < 0.05, ** *p* < 0.01, *** *p* < 0.001.

**Table 3:** Correlations among primary study variables.

		PDS	Tried Drinking	Heavy Drinking	Drunken Behavior
Boys	PDS	-	0.15 ***	0.18 ***	0.10 **
	Father rejection	0.04	0.12 **	0.15 ***	0.11 **
	Father emotional warmth	−0.02	−0.14 ***	−0.13 ***	−0.09 *
	Father over-protection	0.02	0.04	0.03	0.01
	Mother rejection	0.04	0.12 **	0.16 ***	0.14 ***
	Mother emotional warmth	−0.03	−0.14 ***	−0.16 ***	−0.11 **
	Mother over-protection	0.03	0.05	0.05	0.03
Girls	PDS	-	0.18 ***	0.13 **	0.10 **
	Father rejection	0.03	0.20 ***	0.13 **	0.12 **
	Father emotional warmth	−0.01	−0.15 ***	−0.07	−0.07
	Father over-protection	0.06	0.19 ***	0.09 *	0.13 **
	Mother rejection	0.11 **	0.32 ***	0.22 ***	0.23 ***
	Mother emotional warmth	−0.02	−0.21 ***	−0.12 **	−0.11 **
	Mother over-protection	0.12 **	0.25 ***	0.13 **	0.17 ***

Note: PDS, the total score of pubertal development scale. * *p* < 0.05, ** *p* < 0.01, *** *p* < 0.001.

**Table 4:** Regression analyses testing parenting styles as moderators of the relation between pubertal timing and drinking behavior.

		Variable	Β	R^2^ Change
	Step 1	PDS	0.12 **	0.09 ***
Boys		FEW	−0.13 ***	
	Step 2	PDS × FEW	−0.08 *	0.01 *
	Step1	PDS	0.12 **	0.10 ***
		MR	0.16 ***	
	Step 2	PDS × MR	0.08 *	0.01 *
	Step1	PDS	0.13 ***	0.10 ***
		MEW	−0.15 ***	
	Step 2	PDS × MEW	−0.07 *	
Girls	Step 1	PDS	0.16 ***	0.15 ***
		MR	0.33 ***	
	Step 2	PDS × MR	0.10 **	0.01 **
	Step 1	PDS	0.19 ***	0.08 ***
		MEW	−0.22 ***	
	Step 2	PDS ×MEW	−0.08 *	0.01 *
	Step 1	PDS	0.16 ***	0.09 ***
		MOP	0.23 ***	
	Step 2	PDS ×MOP	0.08 *	0.01 *

Note: PDS, the total score of pubertal development scale; FEW, father emotional warmth; MR, mother rejection; MEW, mother emotional warmth; MOP, mother over-protection. * *p* < 0.05, ** *p* < 0.01, *** *p* < 0.001.

## Data Availability

The data presented in this study are available on request from the corresponding author. The data are not publicly available, to protect the teenagers’ privacy.
